# Metabolomic Analysis and Biochemical Profiling of Cadmium-Induced Metabolic Impairment and Its Amelioration by Resveratrol

**DOI:** 10.3390/bioengineering11111141

**Published:** 2024-11-13

**Authors:** Kainat Ilyas, Kanwal Rehman, Hajra Iqbal, Amjad Hussain, Muhammad Sajid Hamid Akash, Mudassar Shahid, Bushra Sadaf

**Affiliations:** 1 Department of Pharmaceutical Chemistry, Government College University, Faisalabad 38000, Pakistan; 2 Department of Pharmacy, The Women University, Multan 66000, Pakistan; 3 Department of Chemistry, University of Okara, Okara 56300, Pakistan; 4 Department of Pharmaceutics, College of Pharmacy, King Saud University, Riyadh 11451, Saudi Arabia; 5 Department of Neurology, University of Minnesota, Minneapolis, MN 55455, USA

**Keywords:** LC-MS/MS, cadmium, lipid metabolites, amino acid metabolomes, resveratrol, ICP-OES, zinc, calcium

## Abstract

Exposure to heavy metals, particularly cadmium (Cd), poses significant health risks because of their toxic effects and potential for bioaccumulation in living organisms. This study examined the biochemical and metabolomic changes induced by Cd exposure in an animal model via advanced liquid chromatography with tandem mass spectrometry (LC-MS/MS) and biochemical assays to reveal significant disruptions in lipid and amino acid metabolism as well as alterations in key metabolic pathways. Cd exposure led to significant weight loss, hyperglycemia, and insulin resistance, indicating its role in metabolic disorders such as diabetes. The accumulation of Cd in the liver and kidneys, identified via ICP-OES, corresponded with elevated levels of liver (ALT, AST) and kidney (BUN, creatinine) biomarkers, suggesting organ-specific toxicity. At the metabolic level, Cd exposure caused the accumulation of lipid metabolites such as ceramides and sphingolipids, which are associated with insulin resistance and broader metabolic impairments. Amino acid metabolism was also significantly disrupted, with increased concentrations of key amino acids such as phenylalanine, tryptophan, and arginine affecting pathways such as the urea cycle and Krebs cycle. These metabolic disturbances are linked to oxidative stress, systemic inflammation, and impaired glucose regulation, as evidenced by elevated CRP and IL-6 levels. The protective effects of resveratrol (RSV) were clearly demonstrated in this study. RSV treatment ameliorated Cd-induced biochemical and metabolic alterations, as shown by improved glycemic control, restored lipid profiles, and normalized amino acid concentrations. Additionally, RSV significantly reduced inflammatory markers and improved liver and kidney function, highlighting its antioxidant properties and potential as a therapeutic agent against Cd toxicity. However, RSV did not significantly reduce Cd accumulation in organs, indicating that its protective effects are related to mitigating oxidative damage and metabolic disruption rather than promoting Cd excretion. This study enhances our understanding of the molecular mechanisms underlying Cd-induced metabolic impairments and highlights the therapeutic potential of RSV in combating Cd toxicity. These findings underscore the need for further research into heavy metal exposure and its mitigation to protect human health, particularly in areas of environmental contamination.

## 1. Introduction

One heavy metal frequently encountered in the environment is Cd, which poses a threat to many forms of life. Cd, an element found in trace amounts in nature, was discovered in 1817 by Friedrich Stromeyer, a German physician. Lead, mercury, and Cd were identified as the three most dangerous heavy metal contaminants by the UN Global Monitoring Program in 1973 [1]. In 2010, the Expert Committee of the WHO determined a monthly acceptable intake of Cd, which was provisionally set at 25 µg/kg body weight. Cd has a one- to three-year residence duration in soil and a two-year residence period in nearshore sediments. Cd can linger in ocean water for more than 7000 years. Although Cd is naturally present in the Earth’s crust, human activities such as industrial processes, mining, and agriculture can also lead to its release into the environment [2]. Communities located near industrial zones and people working in high-risk jobs like mining and manufacturing are at an elevated health risk due to heavy metal exposure. Environmental contamination through soil, water, and air has raised global public health concerns, especially in low- and middle-income countries, where regulatory protections may be less rigorous. In addition to occupational exposure, cigarette smoking and dietary intake are the primary nonoccupational sources of Cd exposure. Cd has an extremely long biological half-life, often exceeding ten years in most tissues, reflecting its persistence after accumulation [3]. The efficiency of cadmium absorption varies by exposure route; inhalation through respiratory particles is significantly more effective (40–60%) than ingestion through the gastrointestinal tract (5–10%) [4]. Metallothionein plays a crucial role in handling cadmium within cells, aiding its distribution and protecting against its toxic effects. Cadmium forms complexes with metallothionein, which are internalized in proximal tubular cells through receptor-mediated endocytosis. The distribution and accumulation of cadmium in various tissues underscore its potential for long-term health effects and complex interactions with biological systems. Cd exposure poses significant challenges not only to metabolic health but also to the development of effective tissue regeneration strategies in environments contaminated by heavy metals. Understanding the biochemical and metabolic disruptions caused by Cd is crucial for developing bioengineered materials and scaffolds that can promote tissue healing while mitigating the effects of environmental toxins. Metabolomics, an advanced field of study, helps to explore how cadmium impacts various metabolites and metabolic pathways in the body [5].

Resveratrol, recognized for its anti-inflammatory, antioxidant, and antiplatelet properties, was first isolated from white hellebore roots in 1940 and later from *Polygonum cuspidatum* roots in 1963 [6]. Traditional medicine also utilizes pterostilbene, a compound similar to RSV found in red sandalwood, to manage diabetes by activating AMPK, which helps regulate blood sugar levels [7]. RSV plays a protective role against Cd toxicity through multiple mechanisms. First, the potent antioxidant properties of RSV enable it to counteract the oxidative stress induced by Cd exposure, scavenge harmful free radicals, reduce lipid peroxidation, and prevent cellular damage. Moreover, RSV can enhance the body’s endogenous antioxidant defenses, mitigating oxidative damage, particularly in the kidneys, where Cd toxicity is pronounced. By regulating various metabolic pathways and cellular processes, RSV has emerged as a promising therapeutic agent for mitigating the adverse effects of Cd at both the organ and systemic levels [8].

This study employed an animal model to explore the metabolomic and biochemical changes caused by Cd and how it dysregulates various metabolic pathways, with a specific focus on lipid and amino acid metabolism. Through metabolomics analysis and biochemical profiling, this study aimed to elucidate the molecular mechanisms underlying Cd-induced metabolic disturbances and to assess the potential for mitigating these effects with RSV intervention. The Cd concentrations in various organs, urine, and serum were also determined via inductively coupled plasma optical emission spectroscopy (ICP-OES), and the organ at greatest risk of Cd accumulation was determined. Given that Cd and zinc compete for the same binding sites, this study explored the correlation between Cd and other essential metals, such as Zn^+2^ and Ca^+2^. This study aimed to improve therapeutic strategies to reduce the impact of metabolic disorders and improve our understanding of how Cd affects metabolic disruption.

## 2. Materials and Methods

### 2.1. Cadmium Solution

A fresh solution was prepared daily throughout the study. To make this solution, 5 mg/kg (obtained from Sigma-Aldrich, St. Louis, MO, USA) was dissolved in 14 mL of purified water, and 0.5 mL of this solution was administered to individual mice via oral gavage.

### 2.2. Resveratrol Solution

RSV, sourced from Chem-Impex Int’l, Inc. (Wood Dale, IL, USA), was obtained as a coarse powder. We dissolved it in corn oil because it is not soluble in water and administered it via oral gavage on the basis of the body weights of the mice.

### 2.3. Study Design

The study involved 48 healthy Swiss albino inbred mice (*Mus musculus*) over a 28-day period, sourced from the animal facility at the Faculty of Pharmacy, GCUF, Pakistan, with ETHICAL approval under reference number 376. The mice, which were nine weeks old and weighed 30 ± 5 g, were housed in stainless steel cages and allowed a one-week acclimatization period in a well-ventilated, air-conditioned room with 40% ± 15% humidity, a 25 ± 5 °C temperature, and a 12 h light/dark cycle. They were provided with a pellet diet and unlimited water. The mice were randomly assigned to four groups of 12: CONT, CdCl_2_, CdCl_2_ + RSV, and RSV. Every week, the serum glucose levels and body weights were recorded. At the end of the treatment period, the mice were fasted overnight, anesthetized, and euthanized by cervical dislocation. Their liver, kidneys, femur bone, heart, lungs, sex organs (testis), and urine were collected, and blood was obtained for serum and plasma separation through cardiac puncture into EDTA-coated tubes.

Group 1: Control group

This group was given normal saline only.

Group 2: Cd group

The Cd-exposed group was administered a solution of CdCl_2_ (5 mg/kg) in purified water by following a method described previously [9] with some modifications.

Group 3: Cd+RSV

In this group, the mice were initially exposed to CdCl_2_ (5 mg/kg) and subsequently treated with RSV (8 mg/kg) dissolved in corn oil via oral gavage one week after Cd administration [10].

Group 4: RSV/Reference group

This group was treated with RSV in corn oil (8 mg/kg) by oral gavage [10].

### 2.4. Measurement of Glycemic Index Parameters

Blood glucose levels were assessed at the beginning and end of the study and every week on Thursday via an Evocheck glucometer to examine the correlation between Cd exposure and diabetes mellitus (DM). Glucose measurements were taken while the participants were in a fasting state. To obtain blood glucose readings, the tail of each mouse was first cleaned with an alcohol swab and then pricked with a needle to collect a blood drop, which was applied to a glucose strip inserted into the glucometer. After a five-second wait, the glucometer recorded the glucose level (mg/dL) for statistical analysis. Additionally, serum insulin levels were measured via an insulin ELISA kit following the manufacturer’s protocols and instructions.

Homeostatic model assessment for insulin resistance (HOMA-IR) scores was calculated from the measured fasting insulin (μLU) and glucose (mg/dL) levels via the following formula:

HOMA IR = Fasting insulin (μLU/mL) × Fasting glucose (mg/dL)/405

Hemoglobin A1c (HbA1c) levels were measured via an HbA1c ELISA following the manufacturer’s instructions.

### 2.5. Assessment of Liver Biomarkers

At the end of the experimental period, the levels of liver function biomarkers aspartate aminotransferase (AST) and alanine transaminase (ALT) in the serum were assessed via an assay kit (Bioactiva Diagnostic, Bad Homburg vor der Höhe, Germany), with absorbance measurements taken via a MicroLab-300 chemistry analyzer.

### 2.6. Estimation of Kidney Function Biomarkers

In accordance with the guidelines in the user manual for the blood urea nitrogen (BUN) and creatinine assay kit (PARS Biochem, Nanjing, China), the serum levels of BUN and creatinine were measured via the sandwich ELISA technique.

### 2.7. Estimation of Inflammatory Biomarkers

At the end of the treatment period, the levels of inflammatory mediators such as C-reactive protein (CRP) and interleukin-6 (IL-6) in the serum were quantified via appropriate ELISA kits from PARS Biochem, China.

### 2.8. Estimation of the Lipid Profile

To assess the effects of heavy metal exposure on dyslipidemia, serum samples were analyzed for key lipid biomarkers, including high-density lipoprotein (HDL), low-density lipoprotein (LDL), cholesterol, and triglycerides (TGs), via their respective assay kits (PARS Biochem, China).

### 2.9. Metabolomic Analysis via LC-MS/MS

Serum samples from mice treated with either Cd alone or Cd combined with RSV were analyzed via LC-MS/MS for metabolomics.

#### 2.9.1. Sample Preparation for LC–MS/MS Analysis

Serum was prepared by centrifuging the blood samples at 3500× *g* for 10 min at 4 °C, and the serum was collected and frozen at −80 °C prior to metabolomics analysis. Proteins were precipitated by adding 10 μL of serum to methanol and incubating at room temperature for 10 min. The supernatant was obtained after recentrifugation at 16,000× *g* for 15 min. After a modified version of a previously established sample preparation method, the supernatant was dried to dryness under a stream of nitrogen gas and reconstituted in 20 μL of pure methanol [11]. For the metabolomic analysis, 10 μL of the reconstituted supernatant was injected into the LC-MS/MS system.

#### 2.9.2. Conditions of the Instrument

The instrument was operated according to the specified parameters outlined in Table S1. An Agilent 6495C triple quadrupole LC-MS system equipped with an electrospray ionization (ESI) source was used. A 2 µL sample was injected into an Agilent Zorbax Extend-C18 Rapid Resolution HT column (2.1 × 100 mm; 1.8 µm). An inline filter with 2 µm frits (Agilent Technologies, CA, USA) was placed just before the analytical column. Chromatographic separation was achieved via a gradient with 0.1% formic acid in water as mobile phase A and 0.1% formic acid in acetonitrile as mobile phase B. The gradient started at 2% B, increasing to 20% B over the first 6 min, then to 45% B from minute 6 to minute 9, and finally to 98% B until 14 min had passed. The flow rate was set at 0.35 mL/min, and the column temperature was maintained at 35 °C. Data were collected in positive electrospray ionization mode (ESI+) with a capillary voltage of 3 kV. The *m/z* range covered was 50–1000. This broad range was divided into multiple segments, with each segment scanned individually to produce several narrow-range spectra during LC-MS/MS analysis. Additional instrumental and operational details are provided in Table S1.

#### 2.9.3. Analysis of Metabolomes by LC-MS/MS

Data were collected via LC-MS/MS in positive ion mode at a capillary voltage of 3 kV. The mass-charge ratio was set within the data gathering range of 50–1000. Those that showed peak compounds were chosen for fragmentation via collision-induced dissociation at energy levels between 20 and 30. When the peaks were selected, the exact molecular masses of the target compounds, as well as their fragmentation patterns, were considered. Validation was achieved through cross-referencing literature sources with precursor ion peaks and their corresponding daughter peaks. This approach has been effective in the identification of several metabolites, including lipid metabolites and amino acids.

### 2.10. Estimation of Cadmium in Serum, Urine, and Organs via ICP-OES

#### 2.10.1. Reagents and Standard Solutions

All reagents were of suprapure quality and were provided by Merck (Darmstadt, Germany). Strictly pure deionized water was used at all levels in this experiment. Ultratile purity grade HNO_3_ was obtained from Sigma-Aldrich. To carry out the ICP-OES analysis, working standards were prepared using 1000 ppm standard solutions of each element supplied by Perkin Elmer, Shelton, CT, USA. All other chemicals were of analytical grade. All the glassware, plastic tubes, and autosampler cups were precleaned with 10% *v*/*v* HNO_3_ for 24 h before distilled water was used for rinsing. All the manipulations were carried out in a clean bench environment to ensure minimal risks from ambient air and dust. The solutions were kept in polyethylene tubes.

#### 2.10.2. Sample Preparation for ICP-OES Analysis

The concentrations of Cd and the essential metals were measured via ICP-OES. The urine samples were collected in metal-free polypropylene containers via a clean catch method and stored until analysis. Prior to experimentation, the urine was filtered. Serum samples and organ tissues were thawed at room temperature. Various tissue samples with different sample weights (Table S2), 1 mL of blood, and 1 mL of urine were taken. All the samples were treated with 3 mL of HNO_3_ and 1 mL of HClO_4_ in cleaned porcelain beakers and left overnight. Sample preparation followed a previously established method with some modifications [12,13]. The acid-treated samples were then heated on an electric hot plate at 80 °C for 2 to 3 h until they became clear and transparent. The resulting solution was diluted to 25 mL with deionized water, yielding a clear, colorless liquid. This mixture was stored in polyethylene bottles at 4 °C until ICP-OES analysis (Teledyne Leeman Labs Prodigy 7). Blank digestions were also conducted, and normal control samples were treated in the same way. For ICP-OES analysis, samples, including calibration blanks, standards, reagent blanks, and control samples, along with matrix modifiers, were introduced. Calibration was regularly checked by analyzing standards every 10 readings. All procedures were carried out at room temperature (25 °C) following established laboratory protocols.

### 2.11. Histopathological Assessment

Histopathological analysis of kidney tissues was conducted to evaluate the effects of Cd exposure. The therapeutic impact of resveratrol was assessed in relation to Cd exposure. The tissues were dehydrated via a series of alcohol concentrations (70%, 95%, and absolute alcohol) through a standard tissue processor. A mixture of 50% xylene and 50% absolute alcohol was employed to clean the tissues, followed by the infusion of melted paraffin wax at 60 °C. Once prepared, the tissues were stained with hematoxylin and eosin. After the slides were prepared for histopathological examination, photomicrographs were captured via a microscope equipped with a 40× magnification lens.

### 2.12. Statistical Analysis

Various statistical analyses were performed via Graph Pad Prism 5 (Graph Pad Software Inc., La Jolla, CA, USA). Descriptive analysis and one-way ANOVA with Bonferroni post hoc correction were utilized for biochemical testing, whereas two-way ANOVA with Bonferroni post hoc correction was used to assess weight and sugar content at different time intervals. Significant differences between groups were determined with a probability threshold of *p* < 0.05. The graphical data are presented as the means and standard deviations (SDs). For metabolome quantification, the fold change formula was applied via Microsoft Excel 365, and paired t tests were conducted via Graph Pad Prism 9.5.1 (Graph Pad Software Inc., La Jolla, CA, USA). Correlations between Cd and essential metals were determined with Graph Pad Prism 9.5.1, and Pearson correlation was used to calculate *p* values and r values.

## 3. Results

### 3.1. Evaluation of Metabolic Impairment Through Biochemical Analysis in Animal Models

We examined the biochemical changes induced by Cd exposure in animal models, focusing on metabolic impairment indicators such as body weight, the glycemic index, liver and kidney function, and inflammatory markers. These experiments aimed to highlight how Cd disrupts normal metabolic processes and the potential protective effects of RSV treatment. The results showed that Cd exposure significantly disrupted body weight regulation, glucose metabolism, and organ function, leading to increased levels of liver and kidney biomarkers and inflammatory markers. RSV treatment effectively mitigated these harmful effects, indicating its potential as a protective agent against Cd-induced metabolic damage.

#### 3.1.1. Body Weight Changes

**Figure 1:**
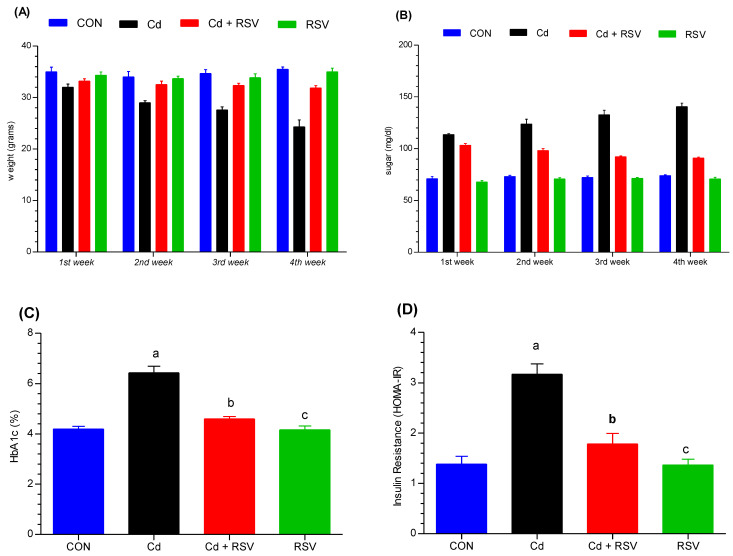
Impact of Cd and treatment on (**A**) weight and (**B**) glucose levels were analyzed via two-way ANOVA followed by the Bonferroni multiple comparison test to evaluate differences across all the data groups. The effects of Cd and treatment on (**C**) HbA1c and (**D**) insulin resistance (HOMA-IR) were assessed via one-way ANOVA followed by Bonferroni post hoc correction. The groups on the x-axis are as follows: CON: control; Cd: cadmium-exposed group; Cd+RSV: cadmium-exposed plus resveratrol-treated group; and RSV: resveratrol-treated group. “a” denotes a significant difference between the Cd-exposed group and the control group. “b” indicates significant differences between the Cd-exposed group and the Cd+RSV-treated group. “c” indicates significant differences between the Cd-exposed group and the RSV-treated group.

All the mice weighed between 30 and 35 g on average before the experiment started. The mice were randomly divided into four different groups: CONT (control), Cd (exposed to cadmium), RSV+Cd (exposed to cadmium with resveratrol), and RSV (resveratrol only). Each mouse in the experiment was given standard chow and water, and throughout four consecutive weeks, their body weights were recorded before every Thursday meal. There was a significant difference in the rate of weight gain between the Cd-exposed group and the control group in the first week (*p* < 0.05). The weight gain of the Cd-exposed group was significantly lower than that of the control group throughout all weeks, with the second, third, and fourth weeks not differing significantly from each other (*p* < 0.001). These trends revealed a decrease in weight gain throughout the treatment period compared with that of the control group. However, interestingly, during the second and third weeks, there was no detectable difference in weight increase between the RSV+Cd group and the control group, suggesting that resveratrol may also somewhat buffer the toxic effects of cadmium. By week four, a significant difference in weight increase was detected between the RSV+Cd group and the control group at *p* < 0.05. Both the CONT and the RSV groups presented the same trends in weight gain throughout the entire trial. The following figure shows these trends in body weight across the different groups (Figure 1A). Compared with the control treatment, Cd exposure led to a significant reduction in weight gain, highlighting its detrimental effect on growth. RSV treatment helped restore normal weight gain patterns, suggesting its protective role against Cd-induced weight loss.

#### 3.1.2. Assessment of Glycemic Index Changes

We investigated the effects of Cd exposure on blood glucose levels, HbA1c, and insulin resistance (HOMA-IR). We measured the basal glucose level of each mouse before the onset of the experiment. Blood glucose levels were measured during the first, second, third, and fourth weeks of the experiment. Compared with those in the Cd+RSV, RSV, and control groups, the blood glucose levels of the mice in the Cd-treated group significantly increased (*p* < 0.001) at the end of the study. No significant differences were found between the Cd+RSV group and the CON or RSV groups. (Figure 1B).

In addition, the values of HbA1c (Figure 1C) and HOMA-IR (Figure 1D) in the CdCl_2_ exposure groups were significantly greater than those in the control and Cd+RSV-treated groups (*p* < 0.05). No significant difference was detected between the RSV or control groups and the Cd+RSV-treated group (*p* > 0.05). The results indicated that Cd exposure significantly increased blood glucose, HbA1c, and HOMA-IR, indicating an elevated risk of diabetes. RSV treatment substantially improved glycemic control, reducing these biomarkers to near-normal levels.

#### 3.1.3. Effects on Liver, Kidney, and Inflammatory Biomarkers

**Figure 2:**
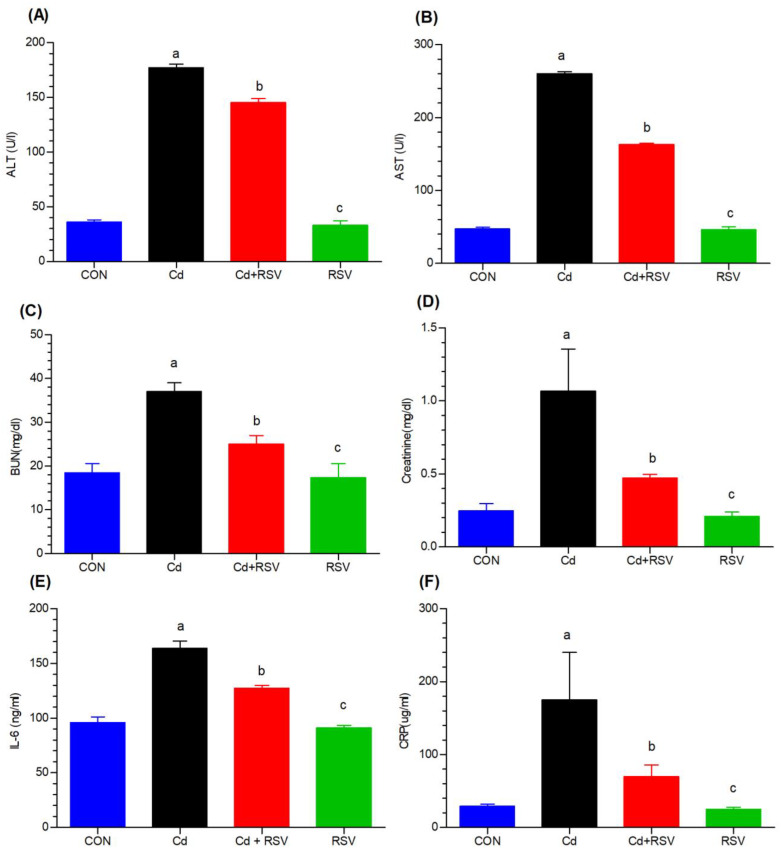
Effects of Cd and treatment on (**A**) alanine transaminase (ALT), (**B**) aspartate aminotransferase (AST), (**C**) blood urea nitrogen (BUN), (**D**) creatinine, (**E**) interleukin-6 (IL-6), and (**F**) C-reactive protein (CRP). The groups on the x-axis are as follows: CON: control; Cd: cadmium-exposed group; Cd+RSV: cadmium-exposed plus resveratrol-treated group; and RSV: resveratrol-treated group. Significant differences were assessed via one-way ANOVA followed by Bonferroni post hoc correction. “a” denotes a significant difference between the CdCl_2_-exposed group and the control group. “b” indicates significant differences between the CdCl_2_-exposed group and the Cd+RSV-treated group. “c” indicates significant differences between the CdCl_2_-exposed group and the RSV-treated group.

The effects of Cd exposure on liver and kidney function were assessed by measuring biomarkers such as ALT, AST, BUN, and creatinine. Additionally, the levels of inflammatory markers (CRP and IL-6) were evaluated to understand the inflammatory response induced by Cd and the protective potential of RSV. Compared with the control group, the Cd-exposed group presented significantly greater levels of blood ALT (Figure 2A) and AST (Figure 2B); significant differences (*p* < 0.05) were noted between the Cd-treated group and the control (CON) and Cd+RSV groups. Compared with the control group, the Cd-exposed group presented significantly greater blood urea (Figure 2C) and serum creatinine (Figure 2D) levels (*p* < 0.001). A significant difference (*p* < 0.01) in the BUN and creatinine levels was also detected between the Cd group and the Cd+RSV group. The concentrations of kidney biomarkers in the Cd+RSV group were comparable to those in the control group, indicating that RSV is a good remedy for reversing Cd-induced damage to kidney function (*p* > 0.05). The inflammatory biomarkers CRP (Figure 2F) and IL-6 (Figure 2E) were also significantly greater in the Cd-exposed group than in the control group, with increasing CRP (*p* < 0.01) and increasing IL-6 (*p* < 0.0001), indicating that CdCl_2_ is important in causing inflammation and metabolic disorders. Compared with Cd exposure alone, treatment with RSV significantly decreased the levels of these inflammatory markers (*p* < 0.05), indicating the anti-inflammatory property of RSV (Figure 2). Compared with those in the control (CON) and RSV groups, the inflammatory marker levels in the RSV-treated group were decreased, and the results were comparable (*p* > 0.05). Cd exposure caused significant liver and kidney damage, as indicated by elevated ALT, AST, BUN, and creatinine levels. The levels of inflammatory markers (CRP and IL-6) were also elevated. However, RSV treatment had a protective effect by reducing the expression of these markers, underscoring its anti-inflammatory and organ-protective effects.

#### 3.1.4. Effects on Lipid Biomarkers

**Figure 3:**
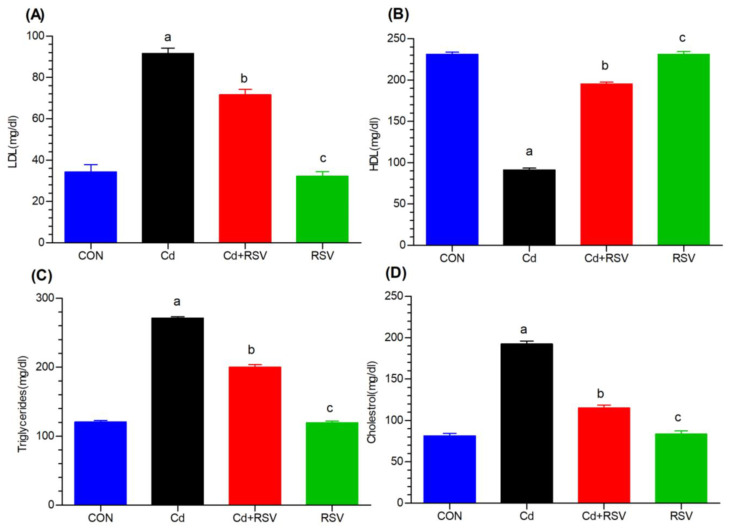
Effects of Cd and treatment on (**A**) low-density lipoprotein (LDL), (**B**) high-density lipoprotein (HDL), (**C**) triglyceride, and (**D**) cholesterol. The groups on the x-axis are as follows: CON: control; Cd: cadmium-exposed group; Cd+RSV: cadmium-exposed plus resveratrol-treated group; and RSV: resveratrol-treated group. Significant differences were assessed via one-way ANOVA followed by Bonferroni post hoc correction. “a” denotes a significant difference between the CdCl_2_-exposed group and the control group. “b” indicates significant differences between the CdCl_2_-exposed group and the Cd+RSV-treated group. “c” indicates significant differences between the CdCl_2_-exposed group and the RSV-treated group.

In this study, we explored the impact of Cd exposure on lipid metabolism, focusing on LDL, HDL, triglyceride, and cholesterol levels. The findings of this study suggest that cadmium exposure is associated with high levels of cholesterol and triglycerides, as well as low-density lipoproteins, in the blood. The mean LDL, triglyceride, and cholesterol serum values were 59.33, 272, and 192, respectively, in the Cd-exposed mice and 34.33, 120.66, and 81.33, respectively, in the control mice. Compared with those in the control group, markedly greater increases in LDL (Figure 3A), triglyceride (Figure 3C), and cholesterol (Figure 3D) levels were detected in the Cd-exposed group (*p* < 0.05). Treatment with RSV greatly reduced cholesterol, triglyceride, and LDL levels to values that were essentially close to those of the control. These findings indicate that RSV plays a preventive role against Cd toxicity. The differences between the Cd+RSV-treated group and the RSV-only group were not significant. Notably, the LDL, triglyceride, and cholesterol levels of the Cd-treated group and the Cd+RSV groups were significantly different. The HDL levels (Figure 3B) of the mice subjected to Cd were also significantly lower than those of the control group (*p* < 0.05). The RSV and Cd+RSV groups presented increased HDL levels; hence, RSV reduces the toxicity levels of Cd. A significant difference was detected (*p* < 0.01) between the Cd and Cd+RSV groups, whereas no differences were detected in HDL between the Cd+RSV and the control or RSV-only groups (Figure 3). Cd exposure resulted in significant dyslipidemia, with increased LDL, TGs, and cholesterol levels, while HDL levels were reduced. RSV treatment helps normalize lipid levels, reinforcing its protective role against Cd-induced lipid metabolism disruptions.

### 3.2. Analysis of Metabolomes by LC-MS/MS

Data were collected using LC-MS/MS in positive ion mode and with a capillary voltage of 3 kV. The mass-to-charge ratio was set within the data gathering range of 50–1000. Those which showed peaked compounds were chosen for fragmentation using collision-induced dissociation at energy levels between 20 and 30. In the case of selecting the peaks, the exact molecular mass of the target compounds, as well as their fragmentation patterns were taken into consideration. Validation was achieved through cross-referencing literature sources with precursor ion peaks and their corresponding daughter peaks. This has been effective in the identification of several metabolites including lipid metabolites and amino acids.

#### 3.2.1. Comparative Analysis of Lipid Metabolites on the Basis of Peak Analysis

We focused on the changes in amino acid metabolomes and lipid metabolites following Cd exposure. The goal of this study was to determine the extent of lipid metabolism disruption and the mitigating effects of RSV. Among those lipid molecules found in both the Cd-exposed group and the RSV-treated group after Cd exposure were sphingolipids, fatty acyls, glycerophospholipids, glycerolipids, and sterol lipids. The lipids were identified via their mass data using internet databases such as LIPID MAPS (http://www.lipidmaps.org/) (accessed on 24 July 2024). The lipid classes identified included diacylglycerols (DGs), triacylglycerols (TGs), ceramides (Cers), glycerophosphocholines (PCs), lysophosphoethanolamines (LPEs), lysophosphatidylcholines (LPCs), glycerophosphoinositols (PIs), sphingomyelins (SMs), and hexosylceramides (HexCers). Various amino acids were identified by matching them with established libraries. Significant differences in the concentrations of certain metabolites were observed between the groups. We compared the Cd-exposed group with the Cd+RSV-treated group to determine the extent of changes in metabolite levels. This comparison was quantified via the fold change formula in an Excel spreadsheet. Compared with those in the Cd+RSV group, metabolites in the Cd-exposed group were upregulated and downregulated, which indicates the disturbance of lipid metabolites (Table 1 and Table 2). Cd exposure caused the upregulation and downregulation of several key lipid and amino acid metabolomes, indicating severe disruptions in metabolic pathways. RSV treatment reversed many of these changes, highlighting its potential to mitigate disturbances caused by Cd.

#### 3.2.2. Metabolome Profiling via Fragmentation Analysis

The ion fragmentation method was used to identify several metabolites. These compounds include sphinganine, lysophosphatidylcholine (LPC 16:0), lysophosphatidylcholine (LPC 11:0), carnitine, phenylalanine, arginine, asparagine, histidine, and tryptophan. The peaks are depicted in two colors: black for the CdCl_2_-treated group and yellowish brown for the Cd+RSV-treated group.

##### Phenylalanine

**Figure 4:**
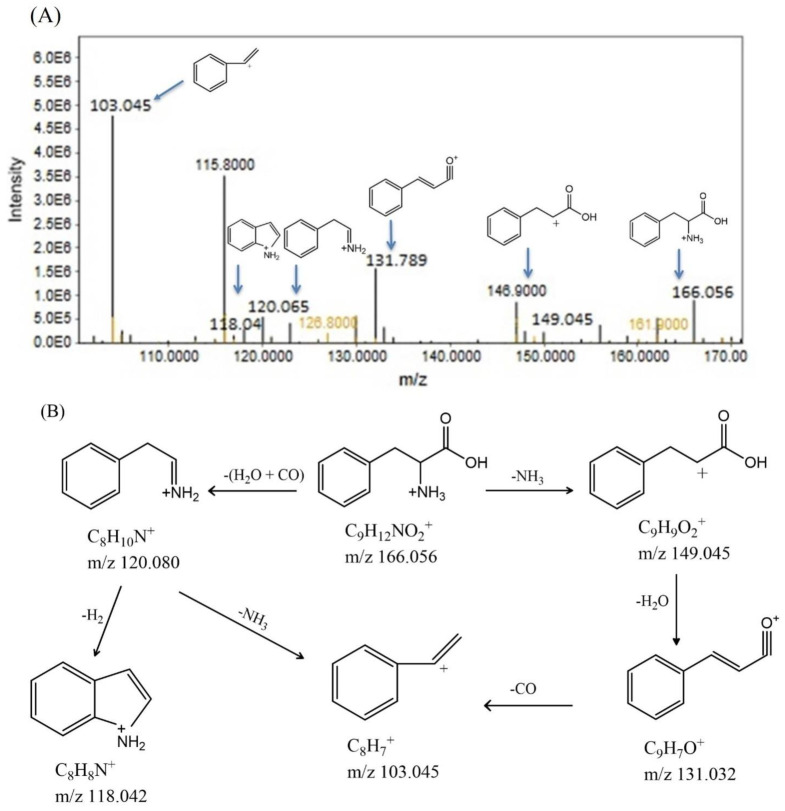
(**A**) shows the precursor ion peaks and fragment ion peaks of phenylalanine in the Cd- and Cd+RSV-treated groups. The black peaks represent the CdCl_2_-treated group, whereas the yellowish-brown peaks represent the Cd+RSV-treated group. (**B**) Fragmentation pattern of phenylalanine.

At a retention time of 3.59 min, the protonated ion of phenylalanine was observed at *m/z* 166.056, which led to the formation of fragment ion peaks at *m/z* 149.045 and 120.065 due to the removal of the NH_3_ and NH_3_+CO groups, respectively. Further elimination of water from the 149.045 fragment resulted in a peak at *m/z* 131.032. This fragment further dissociated, removing CO and producing a peak at *m/z* 103.045, which was also formed by the removal of NH_3_ from the 120.080 fragment. The elimination of H_2_ from the 120.080 fragment produced a peak at *m/z* 118.042. The concentrations of all the peaks that were increased in the Cd-treated group can be found in Figure 4A. The observed fragments are shown in Figure 4B. A peak at *m/z* 166.056 indicated an elevated phenylalanine concentration in Cd-exposed mice. Phenylalanine plays a crucial role in insulin signaling and neurotransmitter synthesis.

##### Arginine

**Figure 5:**
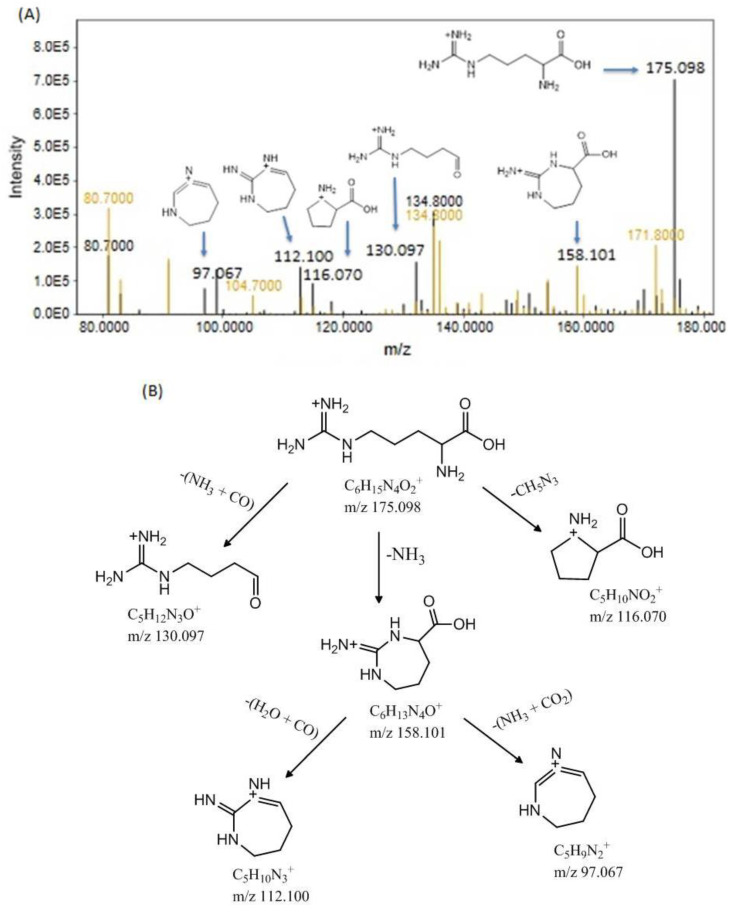
(**A**) shows the precursor ion peaks and fragment ion peaks of arginine in the Cd- and Cd+RSV-treated groups. The black peaks represent the CdCl_2_-treated group, whereas the yellowish-brown peaks represent the Cd+RSV-treated group. (**B**) Fragmentation pattern of arginine.

The molecular mass of arginine is 174.02, and it displays a peak at [M+H] +, *m/z* 175.098, R.T. 3.21 min, as shown in Figure 5A. The peaks are depicted in two colors: black for the CdCl_2_-treated group and yellowish brown for the Cd+RSV-treated group. Upon protonated arginine dissociation, a peak at 116.070 cm^−1^ appeared after the elimination of the guanidine group (CH_5_N_3_). The removal of NH_3_ results in a fragment ion peak at 158.101, which further dissociates into peaks at 112.100 and 97.067 after the removal of H_2_O + CO and NH_3_ + CO_2_, respectively (Figure 5A). Although the same fragment ions formed in both groups, the y-axis intensities of the fragment ions differed notably between the two groups, with higher concentrations observed in the Cd-treated group. The observed fragments are shown in Figure 5B. The increased concentration of arginine in Cd-exposed mice suggests the overactivation of the nitric oxide (NO) synthesis pathway. Excessive NO production disrupts vascular tone, contributes to oxidative stress, and impairs the immune response

##### Asparagine

**Figure 6:**
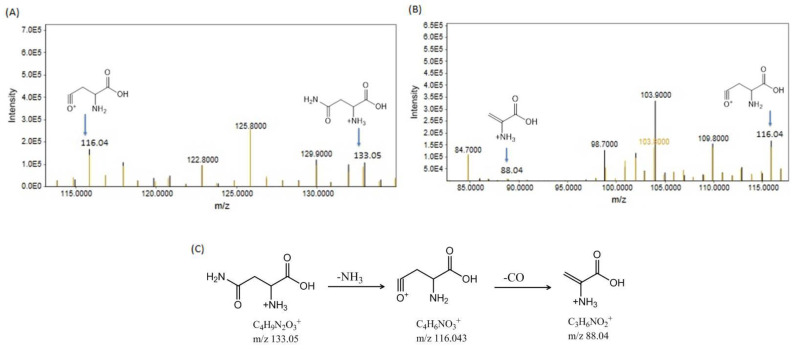
(**A**,**B**) show the precursor ion peaks and fragment ion peaks of asparagine in the Cd- and Cd+RSV-treated groups. The black peaks represent the CdCl_2_-treated group, whereas the yellowish-brown peaks represent the Cd+RSV-treated group. (**C**) The fragmentation pattern of asparagine.

Asparagine, with a molecular mass of 132.12, exhibited a precursor molecular peak at *m/z* 133.05, R.T. 5.10 min. Asparagine exhibited a peak at *m/z* 133.05 after the addition of a proton (H + ion). Following the elimination of NH_3_, a fragment ion peak at *m/z* 116.043 was produced. Further removal of CO from the C_4_H_6_NO_3_^+^ ion resulted in a fragment ion peak at *m/z* 88.04 (Figure 6A,B). The observed fragments are shown in Figure 6C.

##### Histidine

**Figure 7:**
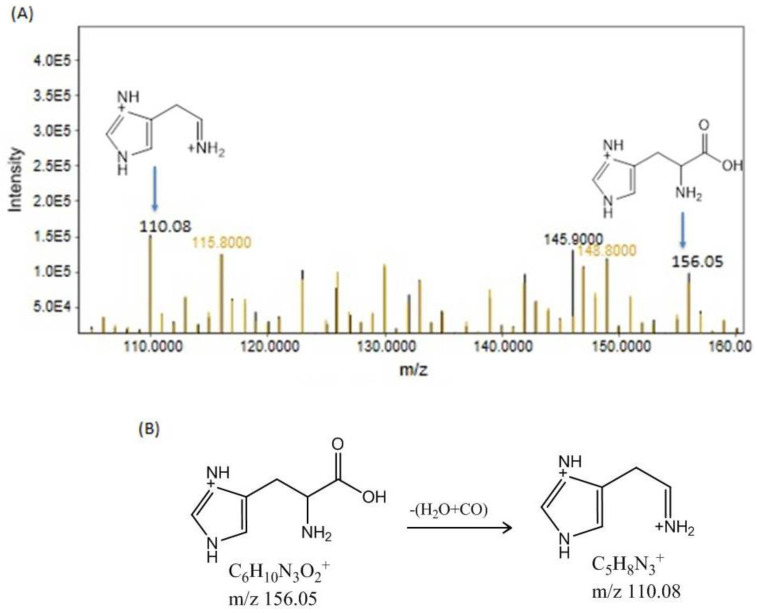
(**A**) shows the precursor ion peaks and fragment ion peaks of histidine in the Cd- and Cd+RSV-treated groups. The black peaks represent the CdCl_2_-treated group, whereas the yellowish-brown peaks represent the Cd+RSV-treated group. (**B**) Fragmentation pattern of histidine.

At R.T. 7.80 min, the precursor ion peak of histidine was observed at *m/z* 156.05 after the addition of a hydrogen ion. This ion subsequently produces a fragment ion peak at *m/z* 110.08 following the removal of H2O and CO (Figure 7A). The observed fragments are shown in Figure 7B. Histidine is the precursor for histamine, which plays a key role in inflammation and immune responses. Elevated histidine concentrations suggest increased histamine synthesis, contributing to the heightened inflammatory response observed in Cd-induced toxicity.

##### Tryptophan

**Figure 8:**
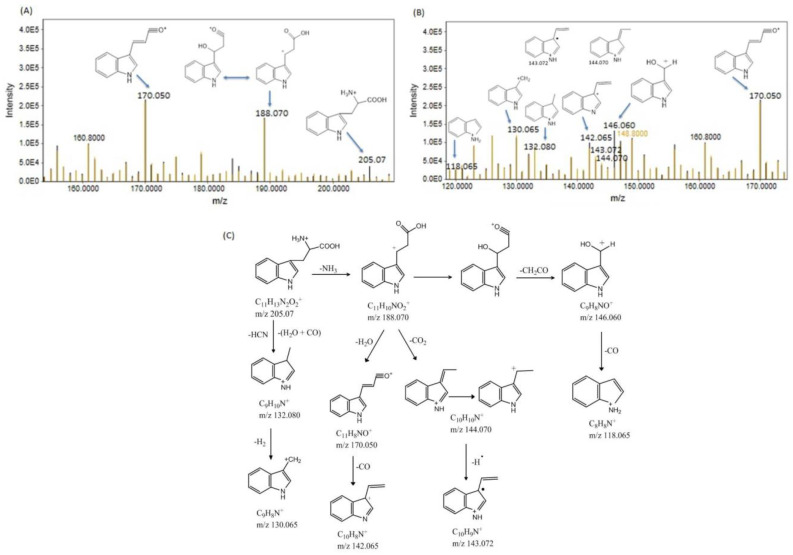
(**A**,**B**) show the precursor ion peaks and fragment ion peaks of tryptophan in the Cd- and Cd+RSV-treated groups. The black peaks represent the CdCl_2_-treated group, whereas the yellowish-brown peaks represent the Cd+RSV-treated group. (**C**) The fragmentation pattern of tryptophan.

At R.T. 8.48 min, tryptophan exhibited a precursor ion peak at 205.07 due to the addition of a hydrogen ion. Subsequent removal of NH_3_ and HCN+H_2_O+CO generated fragment ion peaks at 188.070 and 132.080, respectively. The fragment ion at position 132.080 then underwent the loss of H2 to yield a fragment ion peak at position 130.065. The ion C_11_H_10_NO_2_^+^ underwent further fragmentation, resulting in peaks at *m/z* 146.060 after the loss of CH_2_CO and at 144.070 and 170.050 after the removal of CO_2_ and H_2_O, respectively. The removal of CO from the ions at 146.060 and 170.050 produced fragment ion peaks at 118.065 and 142.065, respectively. Additionally, the ion C_10_H_10_N^+^ produced a radical ion peak at *m/z* 143.072 (Figure 8A,B). The observed fragments are shown in Figure 8C. Elevated tryptophan levels may shift metabolism toward neurotoxic metabolites, potentially contributing to neuroinflammation and cognitive decline. Moreover, tryptophan is essential for serotonin synthesis, and its dysregulation could lead to mood disorders such as depression and anxiety.

##### Sphinganine

**Figure 9:**
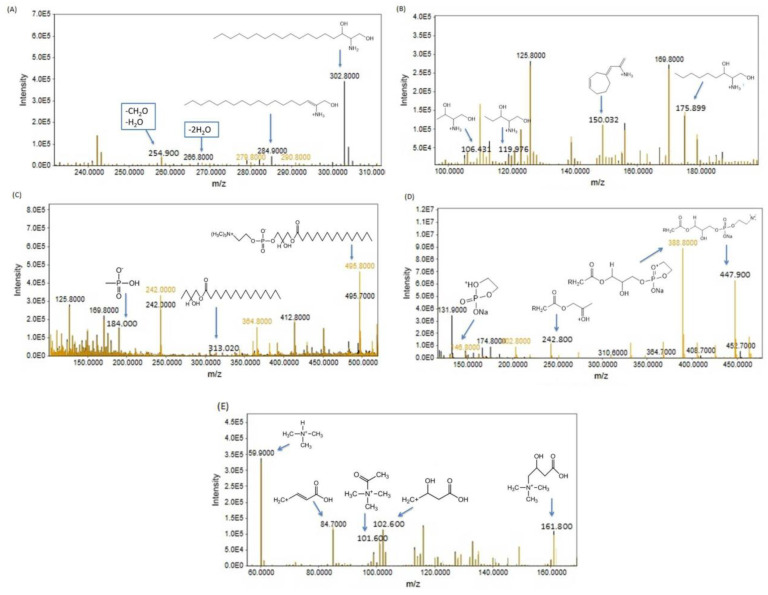
Precursor ion peaks and fragment ion peaks of (**A**,**B**) sphinganine, (**C**) lysophosphatidylcholine (LPC 16:0), (**D**) lysophosphatidylcholine (LGPC 11:0), and (**E**) carnitine in the Cd- and Cd+RSV-treated groups. The black peaks represent the CdCl2-treated group, whereas the yellowish-brown peaks represent the Cd+RSV-treated group.

Sphinganine is a key component of the sphingolipid metabolism pathway. With the chemical formula C₁₈H₃₉NO₂ and a molecular weight of 302, the [M+H] ⁺ ion of sphinganine is detected at 302.8 *m/z*, R.T. 5.93 min. Notable fragmentation peaks include one at 285 *m/z*, resulting from the loss of a water molecule. Further fragmentation revealed a peak at 266.8 *m/z*, corresponding to the removal of two water molecules. Another peak at 254.9 *m/z* was observed after the loss of both water and a CH₂O group. The fragmentation of sphinganine also produced peaks at 175.89, 119.97, and 106.43 m/z, attributed to the loss of alkyl chains containing 9, 13, and 14 carbons, respectively. Additionally, a peak at 150.03 *m/z* was observed following the cyclization of the alkyl chain (Figure 9A,B). The upregulation of sphinganine observed in the cadmium-exposed group was linked to the development of insulin resistance, as evidenced by the HOMA-IR results.

##### Lysophosphatidylcholine (LPC 16:0)

Lysophosphatidylcholine (LPC 16:0), with the molecular formula C₂₄H₅₀NO₇P and a molecular weight of 495.3, has its [M+H]⁺ ion detected at 495.8 *m/z*, R.T 16.74 min. The fragmentation pattern reveals a peak at *m/z* 313.02, corresponding to the alkyl chain, and a peak at *m/z* 184.00, associated with the phosphate group (Figure 9C). In the cadmium-exposed group, the concentration of LPC 16:0 decreased. This reduction in LPC levels can disrupt normal lipid metabolism, leading to lipid accumulation in tissues, a hallmark of type 2 diabetes.

##### Lysophosphatidylcholine (LGPC 11:0)

Lysophosphatidylcholine (LPC 11:0), with the molecular formula C₁₉H₄₀NO₇P and a molecular weight of 425.25, has an [M+Na]⁺ ion at 447.9 *m/z*, R.T. 3.54 min. This is supported by the observation of fragment ions, including a peak at *m/z* 388.8, which results from the direct loss of trimethylamine, as well as peaks at *m/z* 242.8 and 146.8, generated from the dissociation of an intermediate formed through cyclization (Figure 9D). In the cadmium-exposed group, LPC 11:0 also decreased. This reduction can alter the cell membrane composition, which may disrupt insulin receptor function and signaling pathways, ultimately contributing to insulin resistance. A decrease in LPC can increase proinflammatory cytokines, which are known to contribute to the development of insulin resistance and diabetes

##### Carnitine

Carnitine, with the chemical formula C₇H₁₆NO₃ and a molecular weight of 161.105, has its [M+H]⁺ ion detected at 161.8 *m/z*, R.T. 12.24 min. The fragmentation of carnitine results in additional peaks at *m/z* 102.6, 101.6, 84.7, and 59.9, which confirms the presence of carnitine. The removal of trimethylammonium ions from carnitine is indicated by *m/z* 59.9 (Figure 9E). In the Cd-exposed group, carnitine levels were slightly elevated, which may suggest kidney disorders, as impaired excretion can lead to increased concentrations of carnitine.

### 3.3. Detection of Cd by ICP-OES

**Figure 10:**
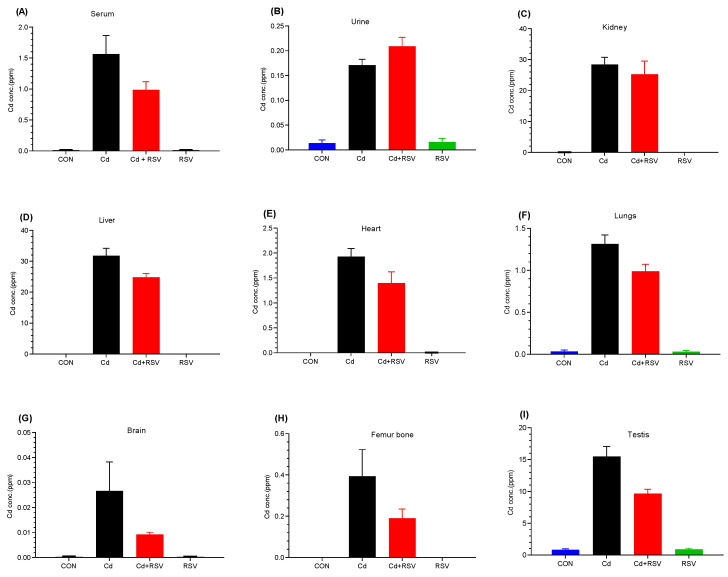
Cd concentrations in various body organs: (**A**) kidney, (**B**) liver, (**C**) serum, (**D**) urine, (**E**) heart, (**F**) lungs, (**G**) brain, (**H**) femur, and (**I**) testis. One-way ANOVA was used to establish significant differences.

We quantified Cd accumulation and evaluated whether RSV affects the distribution and concentration of Cd in the body. Cd concentration was measured in the serum, urine, and various organs, i.e., the kidney, liver, heart, lungs, brain, femur, bone, and testis. Cd accumulates in different organs at different concentrations, with the highest concentrations occurring in the kidney and liver. The mean values observed in the serum (Figure 10A), urine (Figure 10B), kidney (Figure 10C), liver (Figure 10D), heart (Figure 10E), lungs (Figure 10F), brain (Figure 10G), femur bone (Figure 10H), and testis (Figure 10I) of the Cd-exposed group were 1.566, 0.171, 28.43, 31.7, 1.929, 1.316, 0.027, 0.394, and 15.479, respectively. Significant differences (*p* < 0.05) were noted between the Cd-exposed group and the control group and RSV group. However, there was no significant difference observed between the Cd-treated group and the Cd+RSV-treated group. The reason may be that RSV reduces the negative effects of Cd through its antioxidant properties but does not significantly reduce the Cd concentration in various organs, serum, or urine (Figure 10). The results show that Cd accumulates primarily in the kidneys and liver. However, RSV did not significantly reduce the Cd concentration in these organs.

### 3.4. Impact of Cd on Essential Metals

#### 3.4.1. Impact of Cd on Zn Cations

**Figure 11:**
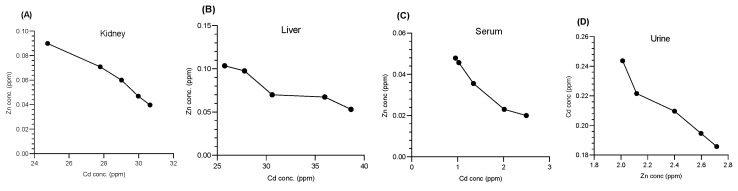
Correlations between the Cd and Zn concentrations in the (**A**) liver, (**B**) kidney, (**C**) serum, and (**D**) urine according to the Pearson correlation. An inverse relationship was found between the concentrations of Cd and Zn ions.

Cd decreases the concentration of Zn ions by competing for the same binding sites. This study examined the correlations between Cd and Zn in the kidney (Figure 11A), liver (Figure 11B), serum (Figure 11C), and urine (Figure 11D). An inverse relationship was found in all the organs, indicating that relatively high Cd levels corresponded to relatively low Zn concentrations. Specifically, the correlation coefficients (r^2^) and *p* values were as follows: kidney (r^2^ = 0.9755, *p* = 0.0016), liver (r^2^ = 0.8848, *p* = 0.0172), serum (r^2^ = 0.9504, *p* = 0.0048), and urine (r^2^ = 0.9496, *p* = 0.0049). (Figure 11). The results indicate a strong inverse correlation between Zn and Cd. Cd exposure led to a significant decrease in Zn levels, demonstrating its ability to disrupt the balance of essential metals.

#### 3.4.2. Impact of Cd on Ca Cations

**Figure 12:**
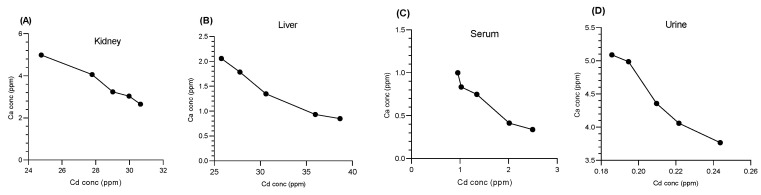
Pearson correlation between the Cd and Ca concentrations in the (**A**) liver, (**B**) kidney, (**C**) serum, and (**D**) urine. An inverse relationship was found between the concentrations of Cd and Ca ions.3.5. Histopathological Changes in the Kidney.

Cd decreases the concentration of Ca ions by interfering with Ca channels. This study examined the correlations between Cd and Ca in the kidney (Figure 12A), liver (Figure 12B), serum (Figure 12C), and urine (Figure 12D). An inverse relationship was found in all the organs, indicating that higher Cd levels corresponded to lower Ca concentrations. Specifically, the correlation coefficients (r^2^) and *p* values were as follows: kidney (r^2^ = 0.9799, *p* = 0.0012), liver (r^2^ = 0.9555, *p* = 0.0040), serum (r^2^ = 0.9450, *p* = 0.0056), and urine (r^2^ = 0.9503, *p* = 0.0048) (Figure 12). All these results indicate a strong inverse correlation between Ca and Cd. Exposure to Cd resulted in a notable reduction in Ca levels, highlighting its potential to disturb the balance of essential metals.

**Figure 13:**
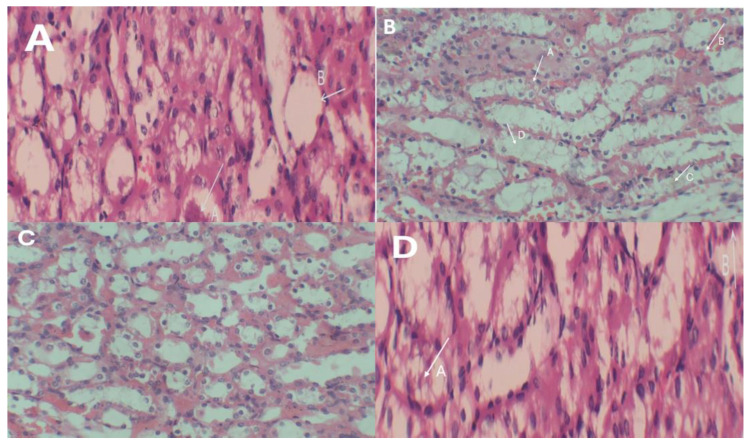
Histology of kidney tissue: (**A**) control group, (**B**) Cd-exposed group, (**C**) group treated with both Cd and RSV, and (**D**) group treated with RSV only.

Figure 13A Kidney tissue exhibits a regular histological arrangement of glomerular and tubular structures without any signs of tissue damage. Figure 13B In contrast, the photomicrograph shows a kidney section from a mouse exposed to Cd orally for 28 days, revealing severe histopathological alterations in kidney tissue. Significant changes include (a) tubular necrosis, (b) vascular degeneration, (c) tubular degeneration, and (d) hydropic degeneration. These findings indicate the harmful effects of Cd exposure on renal tissues. Figure 13C Kidney sections from the group that received RSV treatment after Cd exposure showed mild changes, indicating mild swelling and mild rupturing of kidney tubules, indicating the mitigating effect of resveratrol on cadmium toxicity. Figure 13D The photomicrograph displays a kidney section from a mouse treated solely with RSV. The kidney tissue revealed a histological arrangement comparable to that of the control group, with no disruptions observed in the arrangement of the glomerular and tubular structures. Furthermore, there are no signs of tissue damage.

## 4. Discussion

Cd is a hazardous pollutant that affects multiple organs, including the kidneys, liver, testicles, lungs, and bones [14]. Our research utilized a metabolomics approach to assess how Cd exposure alters biochemical profiles and to evaluate RSV as a protective agent against Cd-induced damage. Cd exposure in mice resulted in significant weight loss, which is consistent with previous studies demonstrating that Cd has a dose- and time-dependent effect on body weight [15]. However, mice treated with both Cd and RSV presented increased body weights, which aligns with findings that RSV can counteract the effects of Cd [16]. Cd exposure is also correlated with increased diabetes incidence, as indicated by increased blood sugar and HbA1c levels [17,18]. RSV has demonstrated potential in managing blood glucose levels and mitigating diabetes, which is supported by the findings of clinical trials showing its effectiveness in improving insulin sensitivity [19]. In our study, the Cd-exposed group presented significant increases (*p* < 0.05) in HbA1c and HOMA-IR compared with those of the control and RSV-treated groups. However, no significant difference was detected between the RSV-treated group and the control group. Previous studies have shown that Cd exposure leads to significant increase in blood glucose levels, while RSV reduces oxidative stress and offers protective effects against diabetes [20].

The nephrotoxicity of Cd is profound, as the kidneys retain approximately 50% of the body’s total Cd, leading to severe effects [21]. Our study revealed that oral Cd administration (5 mg/kg) significantly altered (*p* < 0.05) blood urea nitrogen (BUN) and creatinine levels. Compared with the Cd-only group, the RSV-treated group presented improvements in these markers, with results comparable to those of the control and RSV-only groups. Similar findings were reported in rats, where Cd exposure reduced kidney weight and increased the expression of markers of renal dysfunction, although RSV ameliorated these effects [22]. Various studies have shown that natural substances such as RSV can alleviate Cd-induced nephrotoxicity [23]. This effect was observed in the histopathological findings of the kidney tissue of Cd-exposed mice. Histopathological analysis revealed notable alterations in the kidney morphology of the mice exposed to cadmium. In contrast, those treated with resveratrol exhibited mild signs of healing, indicating its therapeutic potential.

In our study, liver impairment, indicated by elevated AST and ALT levels (*p* < 0.05), was observed in the Cd-exposed group. Cd exposure disrupts liver function, which is correlated with increased liver function markers in the general population [24]. Previous studies have also demonstrated that Cd elevates liver enzyme levels, while RSV has the opposite effect, protecting the liver from Cd-induced hepatic damage [25].

The associated mechanisms include oxidative stress and inflammation, with Kupffer cells and inflammatory cytokines playing significant roles [26]. Counterfactual studies suggest that inhibiting Kupffer cells can reduce Cd-induced liver damage [27]. Cd, by weakening cellular antioxidant defense, exacerbates oxidative stress, which negatively impacts long-term cell survival and function, mainly via inducing oxidative stress [28]. Chronic inflammation associated with Cd exposure is linked to various metabolic disorders, including T2DM [29]. RSV is a potent activator of Sirtuin 1 (SIRT1) and deacetylates several transcription factors, including p53, FOXO, and NF-κB, which enhances cellular survival and reduces inflammation. Our study revealed that compared with control mice, Cd-exposed mice presented increased CRP (*p* < 0.01) and IL-6 levels (*p* < 0.0001), indicating substantial oxidative stress and inflammation. Owing to its antioxidant properties, RSV reduced the levels of these inflammatory markers in the RSV+Cd group, demonstrating its protective benefits. A recent meta-analysis highlighted that RSV could lower CRP levels in patients with T2DM [30].

Consistent with these findings, daily consumption of 40 mg of RSV led to significant reductions in IL-6 and CRP in healthy individuals [12]. Under stress conditions such as Cd exposure, NF-κB becomes activated, leading to the production of proinflammatory cytokines (e.g., IL-6). RSV suppresses NF-κB activation by inhibiting the phosphorylation and degradation of IκB, an inhibitor of NF-κB. This prevents NF-κB translocation to the nucleus, reducing the inflammatory response and oxidative stress. RSV also activates the nuclear factor erythroid 2-related factor 2 (Nrf2) pathway, which is a key regulator of antioxidant responses. By increasing Nrf2 activity, RSV increases the cellular antioxidant capacity, protecting cells from the oxidative damage induced by Cd toxicity [30,31,32].

Additionally, Cd exposure significantly increased TGs and LDL levels (*p* < 0.05) compared with those in the control group, disrupting lipid metabolism, as confirmed by previous studies showing similar effects in Cd-exposed animals [33]. A significant difference was observed between the cadmium-exposed group and the RSV-treated group [34]. Treatment with RSV significantly reduced cholesterol, TGs, and LDL levels to values nearly comparable to those of the control group. These findings suggest that RSV may play a preventive role against cadmium toxicity. Cd accumulation was highest in the kidneys and liver, which aligns with previous research on the tendency of Cd to concentrate in these organs [35,36,37,38,39]. Our study revealed that Cd accumulated primarily in the kidneys and liver, with lower levels detected in other organs and serum. RSV treatment did not significantly reduce the total Cd concentration in these tissues.

This is the limitation revealed by our study: RSV has notable protective effects against Cd-induced oxidative stress and metabolic disturbances, but it did not significantly reduce Cd accumulation in organs. This suggests that RSV may counteract the biochemical and inflammatory effects of Cd toxicity. RSV’s mechanism of action, primarily focused on reducing oxidative damage and modulating metabolic pathways, may not directly interact with Cd-binding proteins.

Physiologically, the accumulation of Cd in critical organs such as the liver and kidneys underscore its long-term toxicity, whereas RSV helps prevent the resulting damage without affecting Cd retention.

In this study, the correlation of Cd with essential metals such as Zn and Ca was also determined, and an inverse relationship was found between Cd and other cations such as Zn and Ca. The reason may be that Cd, Zn, and Ca can interact with overlapping sites within biological systems owing to their chemical properties, but their binding affinities and effects vary. Zinc typically binds to sites involving cysteine residues and thiol groups, playing crucial roles in enzyme function and the immune response through its presence in zinc fingers and other proteins [39]. Calcium, on the other hand, binds to carboxylate groups and negatively charged regions in proteins, which are essential for processes such as muscle contraction, blood clotting, and nerve signaling [40]. Cd, which can mimic zinc due to its similar size and charge, binds to sulfhydryl groups with higher affinity, often displacing zinc and leading to toxicity [41]. While Cd does not directly compete with calcium for binding sites, its toxicity can disrupt calcium homeostasis by affecting calcium channels and signaling pathways. Thus, although these metals can bind to similar sites, their interactions differ significantly, with Cd causing disruption and toxicity compared with the essential roles of zinc and calcium [42]. It can block L-type voltage-gated calcium channels, which are essential for calcium influx into cells, and modulate the activity of other calcium channels and transporters, leading to abnormal calcium flux [43]. Cd also interferes with calcium-binding proteins and affects calcium transporters and pumps, such as calcium ATPases, which are crucial for regulating intracellular and organellar calcium levels [42].

**Figure 14:**
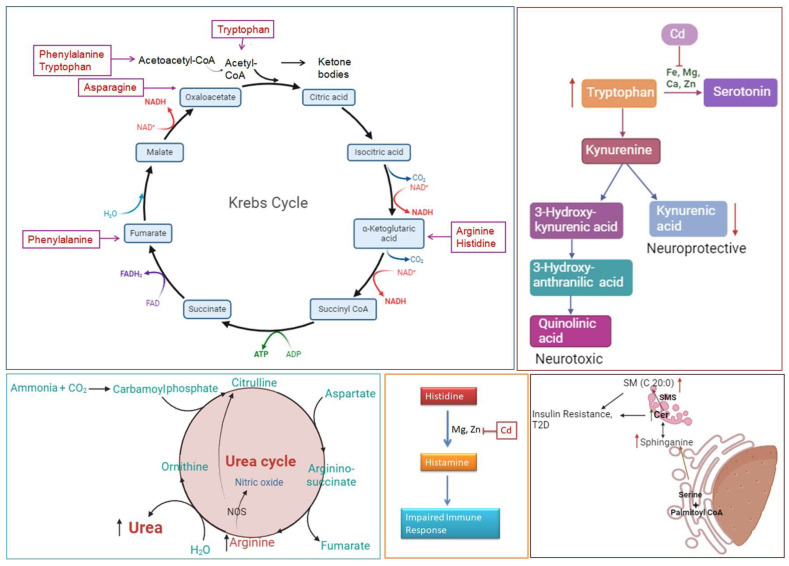
Metabolic pathway disruptions induced by cadmium exposure and their impact on neurotoxicity and metabolic health. This figure illustrates the role of cadmium (Cd) exposure in disrupting key metabolic pathways, with a focus on its neurotoxic effects and adverse impacts on metabolic health. Cd exposure leads to significant alterations in the Krebs cycle, affecting amino acids such as phenylalanine, tryptophan, asparagine, arginine, and histidine, which play essential roles in cellular energy production and metabolism. These disruptions may impair cellular respiration and metabolic efficiency, ultimately influencing overall energy balance. Cd also exerts toxic effects on the tryptophan–kynurenine pathway, redirecting tryptophan metabolism towards increased production of neurotoxic metabolites like quinolinic acid, while reducing levels of neuroprotective metabolites such as kynurenic acid. This imbalance can contribute to neurotoxicity, as quinolinic acid is associated with neuroinflammatory and excitotoxic effects. The pathway’s altered metabolite profile reflects Cd’s neurotoxic potential, which poses risks to neural health and cognitive function. In the urea cycle, Cd exposure impacts the body’s ability to detoxify ammonia, leading to an increase in urea levels. Disruptions in the urea cycle also affect nitric oxide synthesis, potentially compromising vascular and immune functions. Cd further interferes with histamine metabolism by disrupting the conversion of histidine to histamine. This disturbance is partially attributed to Cd’s interference with essential metals like magnesium (Mg) and zinc (Zn), which are critical for immune response regulation. Reduced histamine levels may impair immune response, increasing susceptibility to infections and inflammatory diseases. Lastly, Cd exposure has significant effects on lipid metabolism, particularly through disruptions in sphingolipid and ceramide pathways. Elevated levels of sphingomyelin (SM) and ceramide are associated with insulin resistance and T2DM, suggesting that Cd exposure may contribute to metabolic disorders. By affecting multiple pathways, Cd exposure not only increases neurotoxic risk but also interferes with metabolic processes that are crucial for maintaining health. This figure underscores Cd’s pervasive impact on metabolic and immune health, highlighting the importance of mitigating exposure to this toxic metal.

Compared with those in the RSV-treated group, the concentrations of five amino acids, i.e., arginine, phenylalanine, histidine, tryptophan, and asparagine, were increased in the Cd-exposed group. These findings indicate that RSV has the potential to normalize body pathways. Irregularly increased concentrations of arginine, phenylalanine, histidine, tryptophan, and asparagine in serum can disrupt various biochemical pathways and physiological processes. Elevated arginine levels, for example, can lead to an overactive urea cycle, affecting ammonia detoxification and nitrogen metabolism [44] as shown in Figure 14. The two arginase isoforms, which are crucial for this cycle, require manganese as a cofactor for their activity. However, elevated Cd levels can significantly reduce the availability of essential cofactors such as zinc and manganese, thereby impairing the function of these enzymes and potentially exacerbating metabolic imbalances [45]. Elevated arginine concentrations can also impact the nitric oxide (NO) pathway, leading to excessive production of nitric oxide. This overproduction may disrupt vascular tone, impair the immune response, and alter neurotransmission [46]. Excessive nitric oxide can cause unwanted vasodilation, contributing to changes in blood pressure and vascular function [47]. Research has shown that phenylalanine influences insulin receptor beta (IRβ), leading to reduced glucose absorption and disrupted insulin signaling. Elevated serum levels of phenylalanine in individuals with T2DM have been linked to alterations in insulin receptor function, contributing to insulin resistance and the onset of T2DM symptoms [48,49]. Elevated phenylalanine levels can disrupt multiple biochemical pathways. It can inhibit the conversion of phenylalanine to tyrosine, potentially impairing the synthesis of catecholamines such as dopamine, norepinephrine, and epinephrine, which can negatively impact mood, cognition, and stress responses [50]. Additionally, excessive phenylalanine accumulation is linked to phenylketonuria, a condition characterized by toxic effects and metabolic disturbances, including developmental delays and neurological issues. Essential amino acids such as tryptophan and phenylalanine, which are known to induce insulin resistance, have been linked to an increased risk of both incident and prevalent T2DM [51]. Research indicates that individuals with T2DM have significantly higher levels of tryptophan than controls do. Tryptophan can be metabolized into various physiologically active metabolites via two main pathways: the tryptophan–kynurenine pathway and the tryptophan–methoxy indole pathway. These pathways produce metabolites such as kynurenine, kynurenic acid, and serotonin. Alterations in these metabolic pathways are associated with several pathophysiological processes related to T2DM [52], as shown in Figure 14. Elevated tryptophan levels can significantly impact several biochemical pathways. High tryptophan concentrations may lead to excessive production of serotonin, potentially resulting in mood disorders such as depression or anxiety and affecting sleep, appetite, and pain perception [53]. Additionally, increased tryptophan can shift metabolism toward the kynurenine pathway, which influences neuroinflammation and may contribute to neurodegenerative diseases or altered cognitive function. These metabolic shifts underscore the complex role of tryptophan in both mood regulation and neurological health [54].

Elevated levels of histidine and asparagine, along with other amino acids such as arginine, phenylalanine, and tryptophan, can lead to the abnormal activation of several critical biochemical pathways [55]. Increased histidine can result in excessive histamine production, which may trigger allergic reactions, gastrointestinal problems, and disturbances in the immune response and neurotransmission [56], as shown in Figure 14. Additionally, high histidine levels might affect hemoglobin function and blood cell production, potentially leading to anemia and impaired oxygen transport [56,57]. Overall, irregularly high serum levels of these amino acids can disrupt multiple physiological pathways, contributing to various health issues, including metabolic disorders, mood and cognitive disturbances, immune dysfunction, and cancer progression [58].

In addition to affecting amino acid metabolism, this study highlights an increase in sphinganine levels in Cd-exposed individuals, which may suggest a link to diabetes, as previous research has shown that elevated plasma sphingosine and sphinganine levels correlate with increased ceramide metabolism rates in cells from patients with type 2 diabetes. Ceramides are closely linked to insulin resistance, diabetes, and its complications, including both microvascular and macrovascular effects such as atherosclerosis and diabetic nephropathy. These findings suggest that ceramides could be vital lipid molecules in diabetic patients, particularly in the context of vascular disease, neuropathy, and foot ulcers [59]. Dyslipidemia is a key factor in the onset of insulin resistance. This study revealed a significant decrease in LPC levels. In line with our findings in rodent models, human studies have also shown a widespread reduction in LPC species in the plasma of individuals with obesity and T2DM [60].

LPC offers several protective benefits to the cardiovascular system. It provides vasoprotective effects by modulating the synthesis of prostacyclin and nitric oxide. Specifically, LPC exposure was shown to produce fewer quantities of prostaglandin PGI2 and resulted in endothelial cells that contained more cyclooxygenase-2 and endothelial nitric oxide synthase (eNOS). These findings suggest that LPC can modulate the balance between nitric oxide and endothelin 1. This balance greatly determines the tone of the local blood vessels [61]. Recent studies have shown that HDL becomes enriched with LPC, especially when modified by endothelial lipase, and increases eNOS activity by increasing its presence in the plasma membrane. Additionally, LPC improves the antioxidative capacity of HDL and protects LDL from oxidation [62]. Reduced levels of LPC can lead to disturbances in lipid metabolism, potentially contributing to the development of conditions such as atherosclerosis. This study also revealed disruptions in glycerophosphocholines, which have been linked in the literature to insulin resistance and glucose intolerance [60].

Sphingomyelin (SM C20:0) was found to be upregulated in the Cd-exposed group, which indicated metabolic syndrome. Another previous study revealed that increased blood levels of sphingomyelin (SM) species containing certain saturated acyl chains, such as C18:0, C20:0, C22:0, and C24:0, are strongly associated with key indicators of lipid metabolism, obesity, insulin resistance, and liver function. These findings suggest that these specific SM species could serve as potential biomarkers for metabolic syndrome and related disorders. The elevation of these proteins in the blood may indicate the early onset of metabolic syndrome, providing a link between sphingomyelin profiles and metabolic health [63] as shown in Figure 14. The findings of this study have potential applications in bioengineering, particularly in the design of biomaterials and tissue engineering scaffolds that incorporate bioactive compounds such as RSV to mitigate the effects of heavy metal exposure. The ability of RSV to restore metabolic balance and reduce oxidative stress in Cd-exposed tissues suggests that incorporating such compounds into bioengineered materials could increase tissue resilience and promote regeneration in contaminated environments. Future research could explore how these bioactive materials might be applied in regenerative medicine or environmental bioremediation strategies, offering protection against the toxic effects of environmental pollutants such as cadmium.

## 5. Conclusions

This study provides a comprehensive investigation of the biochemical and metabolomic disruptions caused by Cd toxicity, emphasizing the novel application of advanced metabolomics techniques such as LC-MS/MS to elucidate the complex metabolic pathways affected by Cd exposure. The significant alterations observed in lipid and amino acid metabolism, particularly the dysregulation of sphingolipids, ceramides, and key amino acids such as arginine, phenylalanine, and tryptophan, reveal how Cd disrupts fundamental biological processes involved in energy homeostasis, inflammation, and vascular function. The findings of this study on the metabolic and biochemical disruptions caused by Cd highlight the urgent need for public health interventions to monitor and mitigate Cd exposure, particularly in vulnerable populations. Furthermore, the protective effects of RSV were clearly demonstrated, as RSV not only mitigated Cd-induced damage to metabolic and biochemical pathways but also restored a significant degree of metabolic balance, particularly in the lipid and amino acid profiles. The ability of RSV to counteract the toxic effects of Cd on glucose metabolism, lipid dysregulation, and inflammatory responses highlights its potential as a therapeutic agent, addressing both the immediate toxic effects of Cd and the long-term risks of chronic diseases such as diabetes, cardiovascular disease, and neurodegenerative conditions. In conclusion, this research offers critical insights into the molecular mechanisms underlying Cd-induced metabolic disruptions and highlights the therapeutic potential of resveratrol in mitigating these effects. The findings of this study establish a strong foundation for further research in bioengineering, particularly in the design of biomaterials and tissue engineering scaffolds that incorporate bioactive compounds such as RSV to mitigate the effects of heavy metal exposure. Future studies should explore whether combining RSV with other agents that promote metal detoxification or chelation could provide a more comprehensive approach to reducing Cd toxicity and retention. The ability of RSV to restore metabolic balance and reduce oxidative stress in Cd-exposed tissues suggests that incorporating such compounds into bioengineered materials could increase tissue resilience and promote regeneration in contaminated environments. Future research will explore how these bioactive materials might be applied in regenerative medicine or environmental bioremediation strategies, offering protection against the toxic effects of environmental pollutants such as Cd.

## Figures and Tables

**Table 1:** Changes in amino acid concentrations in the Cd-exposed group compared with those in the Cd+RSV-treated group.

Sr.#	R.T (min)	*m/z*	Metabolite	Regulation	Fold Change	*p* Value	Significance
1	14.917	116.1	Proline	Downregulated	−0.00519	0.621	Non-significant
2	4.199	118.1	Valine	Upregulated	0.78912	<0.001	Significant
3	4.285	132.2	Leucine	Upregulated	6.071174	<0.001	Significant
4	3.483	133.1	L-Ornithine	Upregulated	3.264767	<0.001	Significant
5	14.012	147.2	Glutamine	Upregulated	0.08801	0.040	Significant
6	6.173	197.1	L-Arginine	Downregulated	−0.15066	0.209	Non-significant
7	5.819	205.1	Tryptophan	Upregulated	1.84339	0.006	Significant
8	12.078	263.1	L-Asparagine	Downregulated	−0.12964	0.549	Non-significant
9	4.969	156	Histidine	Upregulated	0.58638	<0.001	Significant
10	20.861	166.1	Phenylalanine	Downregulated	−0.08173	0.226	Non-significant
11	4.852	175.1	Arginine	Upregulated	0.379477	<0.001	Significant

**Table 2:** Changes in lipid concentrations in the Cd-exposed group compared with those in the Cd+RSV-treated group.

Sr.#	R.T (min)	*m/z*	Metabolite	Regulation	Fold Change	*p* Value	Significance
1	3.46	302.5	Sphinganine	Upregulated	0.082136	0.623	Non-significant
2	6.503	326	Cer20:0;O2|Cer 12:0;O2/8:0	Upregulated	0.151338	0.011	Significant
3	6.503	326	CAR 3:0	Upregulated	0.151338	0.011	Significant
4	4.749	338	CAR 12:3	Upregulated	0.507756	0.0002	Significant
5	16.033	496	PC (0:0/16:0)	Downregulated	−0.35501	<0.001	Significant
6	6.802	522.4	LPC 18:1	Upregulated	0.556376	<0.001	Significant
7	4.372	524.3	PE 20:0	Downregulated	−0.1426	0.0006	Significant
8	9.365	560.5	Cer34:1;O2|Cer 12:0;O2/22:1	Upregulated	0.1185	0.0048	Significant
9	8.744	583.3	TG 31:4|TG 8:0_8:0_15:4	Upregulated	0.552885	<0.001	Significant
10	7.659	616.4	PC 24:3|PC 5:0_19:3	Downregulated	−0.37606	<0.001	Significant
11	9.216	627.4	DG 36:10	Downregulated	−0.36206	<0.001	Significant
12	6.794	651.8	DG 37:5	Downregulated	−0.2914	<0.001	Significant
14	4.191	652.4	PC 27:6|PC 5:0_22:6	Upregulated	1.501611	<0.001	Significant
15	5.772	657.4	SM 31:3;O2|SM 12:1;O2/19:2	Downregulated	−0.19907	<0.001	Significant
16	7.533	646.5	SM 20:1;O2/10:0	Upregulated	1.7152057	0.0278	Significant
17	3.106	764.6	AHexCer39:2;O2|AHexCer (O-15:1)12:1;O2/12:0	Downregulated	−0.078	<0.001	Significant
18	4.427	794.7	Cer52:6;O4|Cer 14:1;O3/38:5(2OH)	Upregulated	0.720853	<0.001	Significant
19	2.972	898.7	SL 55:8;O2|SL 19:2;O/36:6;O	Upregulated	0.88367	<0.001	Significant

## Data Availability

All data are available in this manuscript.

## Supplementary Materials

The following supporting information can be downloaded at https://www.mdpi.com/article/10.3390/bioengineering11111141/s1; Table S1: Instrument parameters for LC-MS/MS; Table S2: Weights of the tissue samples.

## Abbreviations

Cadmium: Cd, liquid chromatography with tandem mass spectrometry: LC-MS/MS, inductively coupled plasma-optical emission spectrometry: ICP-OES, calcium: Ca²⁺, zinc: Zn²⁺, resveratrol: RSV, diabetes mellitus: DM, homeostatic model assessment for insulin resistance: HOMA-IR, hemoglobin A1C: HbA1c, aspartate aminotransferase: AST, alanine transaminase: ALT, blood urea nitrogen: BUN, C-reactive protein: CRP, interleukin-6: IL-6, high-density lipoprotein: HDL, low-density lipoprotein: LDL, triglycerides: TGs, electrospray ionization: ESI, diacylglycerols: DGs, triacylglycerols: TGs, ceramides: Cers, glycerophosphocholines: PCs, lysophosphoethanolamines: LPEs, lysophosphatidylcholines: LPCs, glycerophosphoinositols: PIs, sphingomyelins: SMs, hexosylceramides: HexCers, acylcarnitines: CAR.
