# Supplementary material for: Metabolomic Analysis and Biochemical Profiling of Cadmium-Induced Metabolic Impairment and Its Amelioration by Resveratrol
Source: Bioengineering (Basel). 2024 Nov 13;11(11):1141. doi: 10.3390/bioengineering11111141 (PMC11592041; doi:10.3390/bioengineering11111141)
Supplement: Supplementary file 1 [file bioengineering-11-01141-s001.zip › bioengineering-3256012-supplementary.pdf]

Table S1: Instrument parameters for LC–MS/MS.

**Parameters**	**Particulars**
Instrument model	Agilent's 6495C triple quadrupole LC- MS/MS equipped with an electrospray ionization source
Mode of Ion	Positive mode
Mass range	50-1000 m/z
Flow rate	8 L/min
Temperature	300°C
Auxiliary gas flow rate	14/min
Capillary voltage	3000 V
Nozzle voltage	1500 V

Table S2: Weights of the tissue samples.

**Tissue**	**Weight (gm)**
Kidney	0.57 ± 0.02
Liver	0.93 ± 0.06
Lungs	0.72 ± 0.02
Heart	0.50 ± 0.02
Brain	0.54±0.02
Testis	0.10±.02
Femur bone	0.78±0.07
